# Abnormal gut microbiota composition contributes to the development of type 2 diabetes mellitus in db/db mice

**DOI:** 10.18632/aging.102469

**Published:** 2019-11-23

**Authors:** Fan Yu, Wei Han, Gaofeng Zhan, Shan Li, Xiaohong Jiang, Long Wang, Shoukui Xiang, Bin Zhu, Ling Yang, Ailin Luo, Fei Hua, Chun Yang

**Affiliations:** 1 Department of Endocrinology, The Third Affiliated Hospital of Soochow University, Changzhou, China; 2 Department of Neurosurgery, The Third Affiliated Hospital of Soochow University, Changzhou, China; 3 Department of Anesthesiology, Tongji Hospital, Tongji Medical College, Huazhong University of Science and Technology, Wuhan, China; 4 Department of Critical Care Medicine, The Third Affiliated Hospital of Soochow University, Changzhou, China; 5 Department of Cardiology, The Third Affiliated Hospital of Soochow University, Changzhou, China; 6 Department of Anesthesiology and Perioperative Medicine, The First Affiliated Hospital of Nanjing Medical University, Nanjing, China

**Keywords:** gut microbiota, diabetes mellitus, pseudo-germ-free mice

## Abstract

It is well recognized that type 2 diabetes mellitus (T2DM) is an age-related metabolic disease, emerging gradually as a major global health burden that has gained public attention. Meanwhile, increasing attention is paid to the crucial role of gut microbiota in the pathogenesis and therapeutic mechanisms of metabolic disorders, especially T2DM. In this study, we used C57 BL/KS db/db male mice as a T2DM murine model. We found that the β-diversity and relative abundances of gut bacteria were obviously altered in db/db mice, associated with a significant increase in Verrucomicrobia at six levels (phylum, class, order, etc.) and family S24-7 and a significant decrease in Bacteroidaceae at family, genus, and species levels, as well as Prevotellaceae at family and genus levels. Furthermore, fecal bacteria from db/db and m/m mice transplanted into pseudo-germ-free mice showed a significant change in the metabolic parameters, including the body weight, fasting blood glucose, fluid and food intake, and alterations in the composition of the gut microbiota. Taken together, these findings suggest that abnormalities in the composition of the gut microbiota might contribute to the development of T2DM and that potential therapeutic strategies improving gut microbiota might provide beneficial effects for individuals with T2DM and age-related glucose intolerance.

## INTRODUCTION

Aging is highly correlated with a decline in the metabolic rate and glucose intolerance, which might trigger the potential onset of diabetes mellitus [1]. Diabetes mellitus, particularly type 2 diabetes mellitus (T2DM), is emerging as a leading cause of disability and mortality in the aging population [2]. According to reports in the literature, approximately 435 million people were diagnosed with diabetes worldwide and the number is estimated to reach over 642 million by 2040 [3, 4]. Currently, the incidence of T2DM in elderly patients above 65 years of age is greater than 25% [5, 6]. Notably, patients with T2DM, particularly the elderly, exhibit a higher incidence of suffering from microvascular and macrovascular complications [5, 7]. In this regard, the increasing prevalence of diabetes has become a global health concern, which is largely attributed to its inevitable and tricky complications [4, 8].

Recently, an increasing number of studies have focused on the early diagnosis and therapeutic mechanisms of T2DM since T2DM is a chronic disease in the elderly population with an intricate pathogenesis characterized by different levels of genetic susceptibility and environmental risk factors [9–11]. Indeed, individuals with obesity are more likely to suffer from T2DM [12, 13]. It has been reported that systemic inflammation might underlie the pathogenesis of obesity that is, at least partially, related to dysfunctional gut microbiota [14], and reduced microbial diversity in obesity and metabolic dysregulation were obviously detected [15]. It is, therefore, likely that gut microbiota, as a potential environmental factor for energy metabolism, might be closely associated with metabolic disorders [16].

The human gut, which is a complex ecosystem, consists of more than 10^14^ microbes that play a critical role in energy homeostasis, metabolic signaling, and the immune system [17, 18]. It is well recognized that five bacterial phyla predominantly exist in the human gut community: *Actinobacteria*, *Bacteroidetes*, *Firmicutes*, *Proteobacteria*, and *Verrucomicrobia* [19]. Interestingly, Gram-negative bacteria, including *Bacteroidetes* and *Proteobacteria*, were found to be increased in patients with T2DM [20], and they can secrete lipopolysaccharide (LPS) endotoxins, ultimately leading to metabolic endotoxemia. In addition, patients with T2DM as well as obese and overweight subjects, have higher levels of *Bacteroides* and lower levels of *Firmicutes* and *Prevotella* compared with healthy individuals [21, 22], whereas the *Bacteroidetes*/*Firmicutes* ratio as well as the Bacteroides/*Prevotella* ratio was observed to be highly correlated with plasma glucose concentration [23]. There is a causal link between low-grade inflammation and obesity, which is likely related to abnormal and dysfunctional gut microbiota. Dysbiosis might be a trigger for the imbalance in inflammatory responses, finally causing the development of obesity and T2DM. Collectively, an abnormal composition of the gut microbiota might be directly responsible for low-grade inflammation that ultimately acts as a predisposing factor for the progression of obesity and T2DM [22].

In the present study, we adopted C57 BL/KS db/db male mice as a T2DM murine model, and we determined alterations in the gut microbiota using 16S rRNA sequencing. Furthermore, we examined the effects of fecal bacteria transplantation from db/db and m/m mice on pseudo-germ-free mice, assessing the metabolic parameters, including the body weight, fasting blood glucose, and fluid and food intake, and we detected the gut microbiota compositions of the host.

## RESULTS

### Differences in metabolic parameters and gut microbiota profiles between db/db and m/m mice

**Figure 1:**
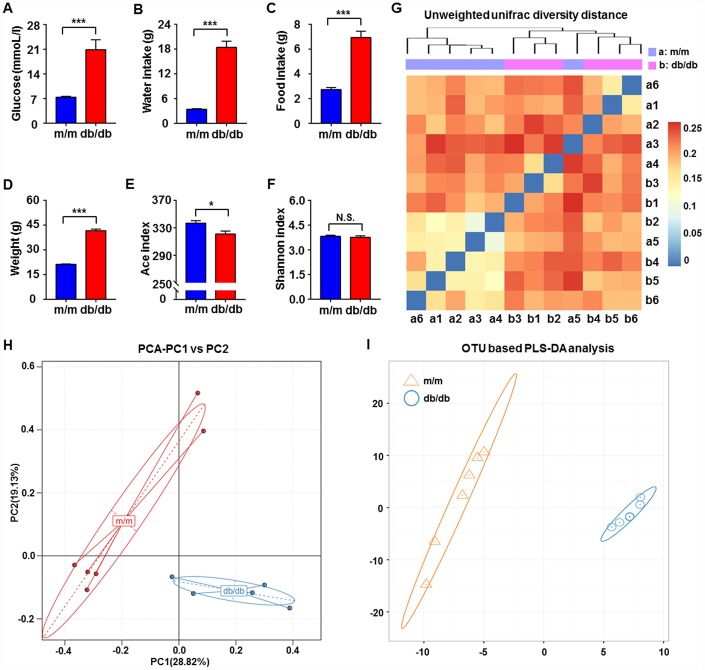
**Comparisons of metabolic parameters and gut microbiota profiles in db/db and m/m mice.** (**A**) Blood glucose (*t*_10_ = 4.762, *P* < 0.001); (**B**) water intake (*t*_10_ = 9.981, *P* < 0.001); (**C**) food intake (*t*^10^ = 7.851, *P* < 0.001); (**D**) body weight (*t*_10_ = 20.41, *P* < 0.001); (**E**) Ace index (*t*_10_ = 2.823, *P* < 0.05); (**F**) Shannon index (*t*_10_ = 0.6029, *P* > 0.05); (**G**) unweighted UniFrac diversity distance; (**H**) PCA analysis of gut bacteria (PC1 versus PC2); (**I**) PLS-DA analysis of the data of gut bacteria. Data are shown as mean ± SEM values (n=6). ^*^P < 0.05, ^**^P < 0.01, ^***^P < 0.001. NS, not significant; PCA, principal component analysis; PLS-DA, partial least squares-discriminant analysis; SEM, standard error of the mean.

It is well acknowledged that db/db mice are a rodent model that is genetically diabetic owing to missense mutations of leptin receptors [24]. In the present study, we adopted db/db mice as a model to study T2DM. Obviously, blood glucose levels, body weight, and water and food intake showed significant increases in db/db mice compared with the control subjects (Figure 1A–1D). We compared the differential composition of gut microbiota between the db/db and control phenotype mice using 16S rRNA analysis of fecal samples. According to the evaluations of Ace, Chao, Shannon, and Simpson indices, α-diversity is commonly linked with species richness in a community or a biotic habitat [25, 26]. The Ace index was found to be significantly lower in the fecal samples of db/db mice compared to m/m mice, although there was no difference in the Shannon index between the two groups (Figure 1E and 1F). Furthermore, β-diversity analysis was used to evaluate the differences of gut microbiota in species complexity [27]. A heat map of β-diversity distance distribution is shown in Figure 1G, a picture of unweighted UniFrac diversity distance. Principal component analysis (PCA) and partial least squares-discriminant analysis (PLS-DA) plots of Bray–Curtis dissimilarity between the two groups showed that the dots of db/db mice were not close to the dots of m/m mice (Figure 1H and 1I). It is, therefore, likely that db/db mice have a distinct profile in gut microbiota composition compared to m/m mice.

### Alterations in gut microbiota composition in db/db and m/m mice

**Figure 2:**
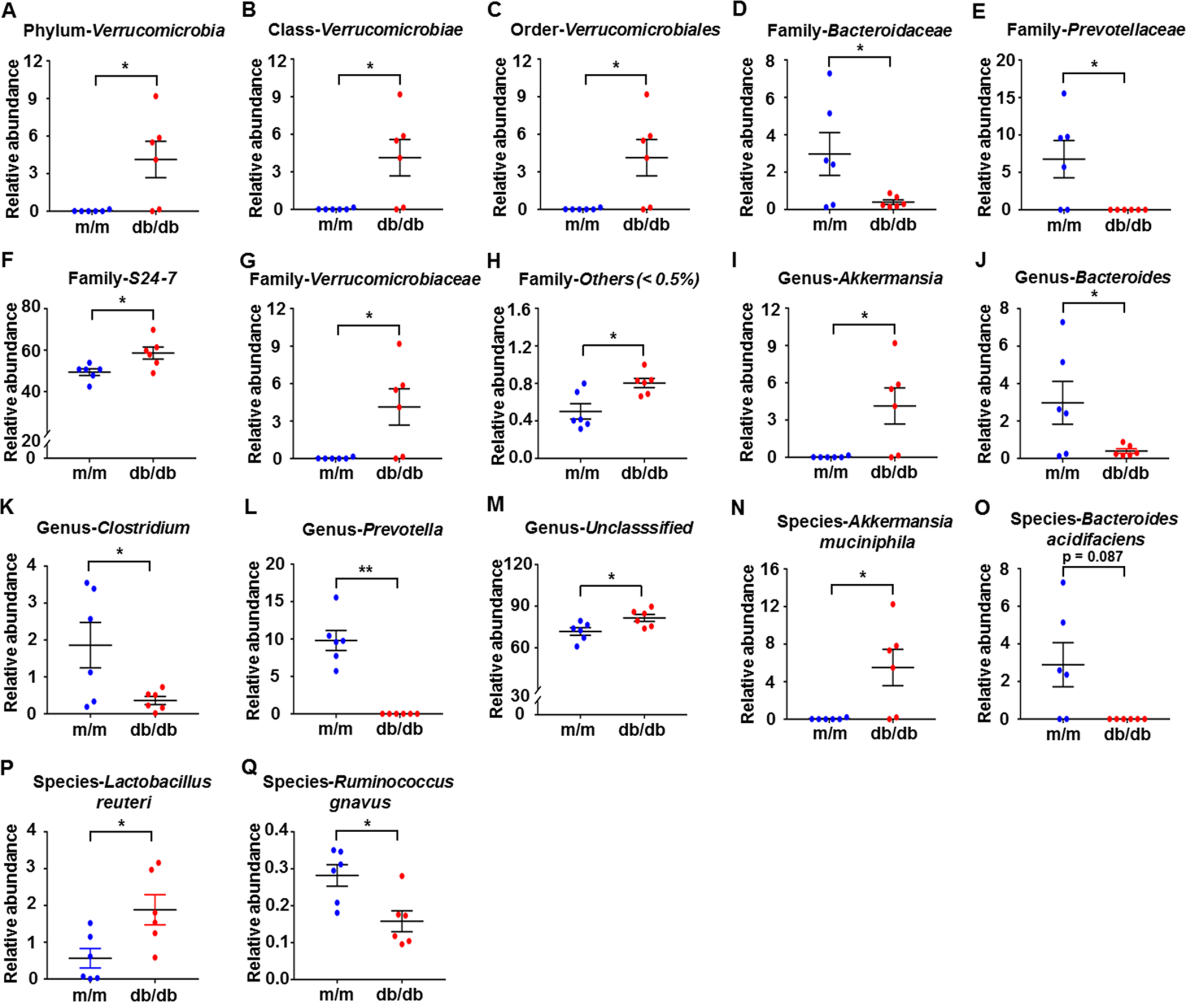
**Differences in the composition of the gut microbiota at the phylum, class, order, family, genus, and species levels between db/db and m/m mice.** (**A**) Phylum *Verrucomicrobia* (Mann–Whitney *U* test; *P* < 0.05); (**B**) Class *Verrucomicrobiae* (Mann–Whitney *U* test; *P* < 0.05); (**C**) Order *Verrucomicrobiales* (Mann–Whitney *U* test; *P* < 0.05); (**D**) Family *Bacteroidaceae* (*t*_10_ = 2.236, *P* < 0.05); (**E**) Family *Prevotellaceae* (*t*_10_ = 2.714, *P* < 0.05); (**F**) Family *S24-7* (*t*_10_ = 2.796, *P* < 0.05); (**G**) Family *Verrucomicrobiaceae* (Mann–Whitney *U* test; *P* < 0.05); (**H**) Family *Others* (<0.5%) (Mann–Whitney *U* test; *P* < 0.05); (**I**) Genus *Akkermansia* (Mann–Whitney *U* test; *P* < 0.05); (**J**) Genus *Bacteroides* (*t*_10_ = 2.236, *P* < 0.05); (**K**) Genus *Clostridium* (*t*_10_ = 2.399, *P* < 0.05); (**L**) Genus *Prevotella* (Mann–Whitney *U* test; *P* < 0.01); (**M**) Genus *Unclassified* (*t*_10_ = 2.62, *P* < 0.05); (**N**) Species *Akkermansia muciniphila* (Mann–Whitney *U* test; *P*<0.05); (**O**) Species *Bacteroides acidifaciens* (Mann–Whitney *U* test; *P* = 0.087); (**P**) Species *Lactobacillus reuteri* (*t*_10_ = 2.708, *P*<0.05); (**Q**) Species *Ruminococcus gnavus* (t_10_ = 3.059, *P*<0.05). Data are shown as mean ± SEM values (n=6). *P<0.05, **P<0.01, ***P<0.001. NS, not significant; SEM, standard error of the mean.

We used 16S rRNA gene sequencing to compare the alterations in the relative abundances of gut microbiota in db/db and m/m mice. The results showed that a total of 17 gut bacteria differed between fecal samples from db/db and m/m mice at six phylogenetic levels (phylum, class, order, family, genus, and species) (Figure 2A–2Q). The relative abundances of 10 gut bacteria were significantly increased in db/db mice compared with m/m mice (Figure 2A–2C, 2F–2I, 2M, 2N, and 2P). On the contrary, the relative abundances of 7 gut bacteria were decreased in db/db mice compared to m/m mice (Figure 2D, 2E, 2J–2L, 2O, and 2Q).

### Abundances of gut microbiota composition at the phylum, class, order, family, genus, and species levels between db/db and m/m mice

**Figure 3:**
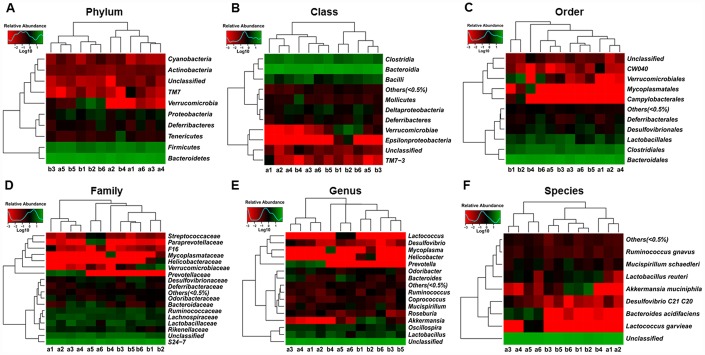
**Heat maps of the composition of the gut microbiota in db/db and m/m mice.** (**A**) Phylum level; (**B**) Class level; (**C**) Order level; (**D**) Family level; (**E**) Genus level; (**F**) Species level.

Heat maps of the gut microbiota composition at the phylum, class, order, family, genus, and species levels between db/db and m/m mice are shown in Figure 3A–3F.

### Effects of db/db and m/m gut microbiota transplantation on metabolic parameters in pseudo-germ-free mice

**Figure 4:**
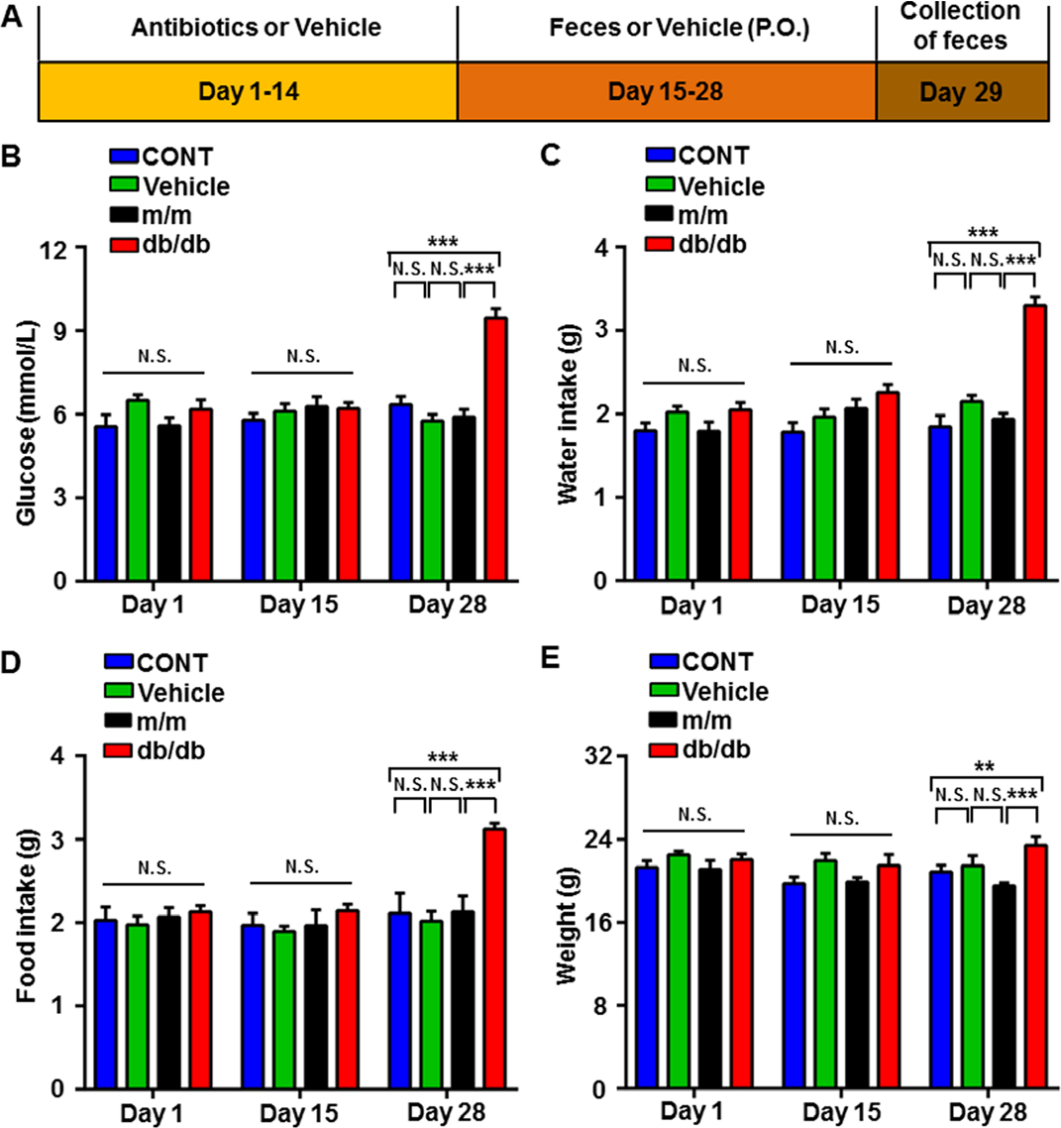
**Changes in the metabolic parameters of pseudo-germ-free mice after fecal transplantation from db/db and m/m mice.** (**A**) Schedule of the present study. Host mice were treated with large doses of antibiotics for 14 consecutive days and were orally treated with fecal microbiota from db/db or m/m mice. Fecal samples were collected for 16S rRNA gene sequencing on day 29. (**B**) Blood glucose (Time: F_2_,_12_ = 16.24, *P* < 0.001; Group: F_3,18_ = 15.42, *P* < 0.001; Interaction: F_6,36_ = 11.33, *P* < 0.001). (**C**) Water intake (Time: F_2,12_ = 26.33, *P* < 0.001; Group: F_3,18_ = 22.17, *P* < 0.001; Interaction: F_6,36_ = 11.74, *P* < 0.001). (**D**) Food intake (Time: F_2,12_ = 13.73, *P* < 0.001; Group: F_3,18_ = 8.878, *P* < 0.001; Interaction: F_6,36_ = 6.519, *P* < 0.001). (**E**) Body weight (Time: F_2,12_ = 5.57, *P* < 0.05; Group: F_3,18_ = 5.319, *P* < 0.01; Interaction: F_6,36_ = 2.03, *P* > 0.05). Data are shown as mean ± SEM values (n=7). *P < 0.05, **P < 0.01, ***P<0.001. CONT, control; NS, not significant; SEM, standard error of the mean.

As shown in Figure 4A, the pseudo-germ-free murine model was induced by large doses of antibiotics that were dissolved in the drinking water for 14 consecutive days. Subsequently, the gut microbiota of the db/db and m/m mice was transplanted into the gastrointestinal tract of the pseudo-germ-free mice for another 14 consecutive days. We measured the metabolic parameters according to previously mentioned procedures. On day 28, the body weight of the db/db mice, fluid and food intake, and fasting blood glucose, showed a significant increase compared to the m/m group or control (CONT) group, although there were no significant changes among the four groups on days 1 and 15 (Figure 4B–4E).

### Effects of db/db and m/m gut microbiota transplantation on α-diversity and β-diversity in pseudo-germ-free mice

**Figure 5:**
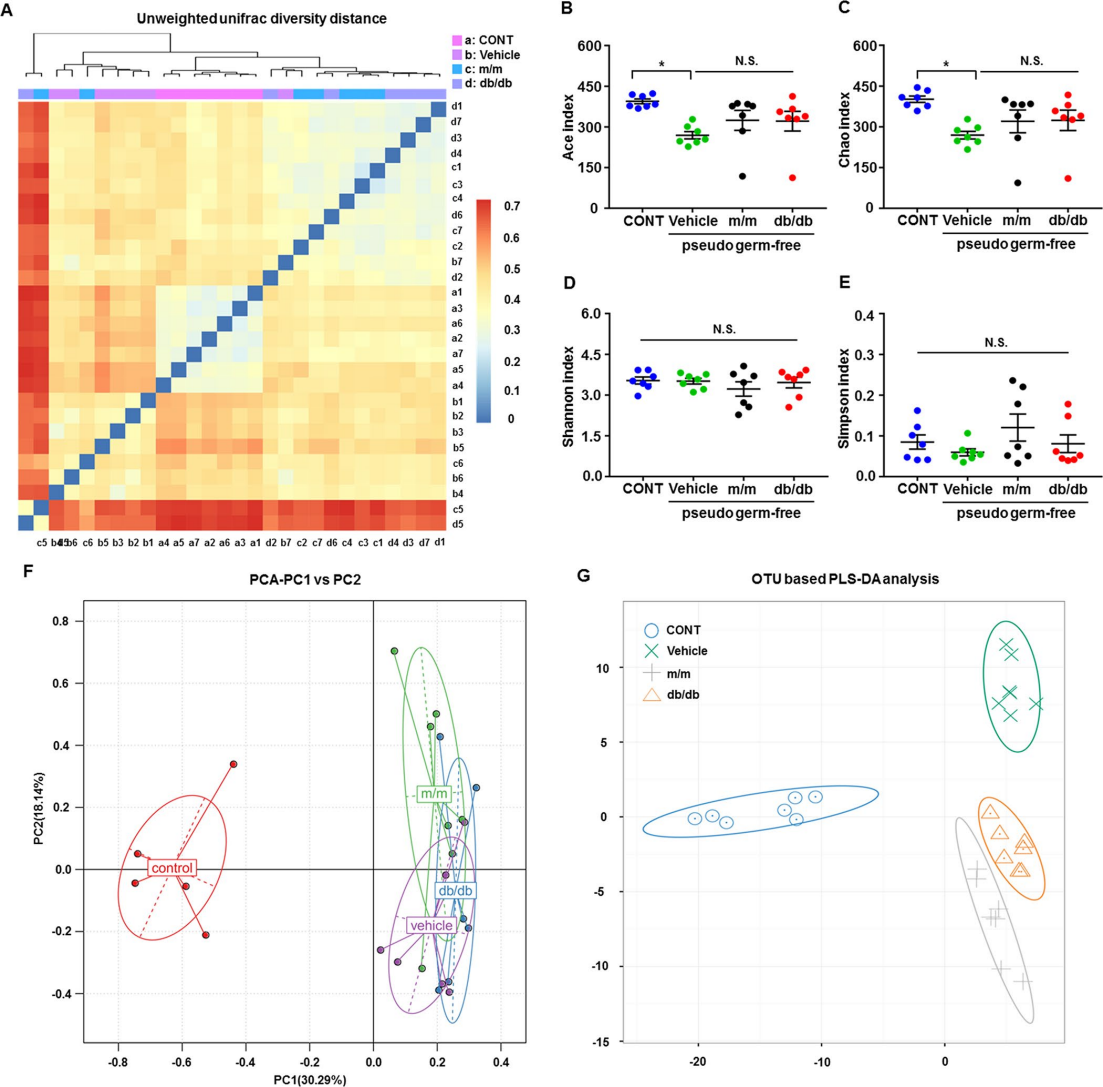
**Alterations in the gut microbiota profiles of pseudo-germ-free mice after transplantation from db/db and m/m mice.** (**A**) Unweighted UniFrac diversity distance; (**B**) Ace index (F_3,24_ = 3.571, *P* < 0.05); (**C**) Chao index (F_3,24_ = 3.415, *P* < 0.05); (**D**) Shannon index (F_3,24_ = 0.6078, *P* > 0.05); (**E**) Simpson index (F_3,24_ = 1.305, *P* > 0.05); (**F**) PCA analysis of the data of gut bacteria (PC1 versus PC2); (**G**) PLS-DA analysis of the data of gut bacteria. Data are shown as mean ± SEM values (n=7). *P < 0.05, **P < 0.01, ***P < 0.001. CONT, control; PCA, principal component analysis; PLS-DA, partial least squares-discriminant analysis; NS, not significant; SEM, standard error of the mean.

After fecal bacteria transplantation in the pseudo-germ-free mice, we analyzed the profiles of gut microbiota among the CONT, vehicle, m/m, and db/db groups (Figure 5A–5G). The picture of unweighted UniFrac diversity distance in Figure 5A suggested a significant difference in the composition of gut microbiota among the CONT, vehicle, m/m, and db/db groups. Meanwhile, vehicle-treated pseudo-germ-free mice had significantly decreased α-diversity indices, including the Ace index and Chao index, whereas the Shannon index and Simpson index failed to show a significant change among the four groups (Figure 5B–5E). Additionally, the PCA plots for the four groups showed that the dots of the control mice were not close to those of mice in other groups, and the plots for the four groups were separated from each other in PLS-DA (Figure 5F and 5G). Thus, it is likely that the effects of fecal microbiota transplantation from db/db and m/m mice to the host gut bacteria were obviously differential.

### Effects of db/db and m/m gut microbiota transplantation on the abundances of host gut microbiota

**Figure 6:**
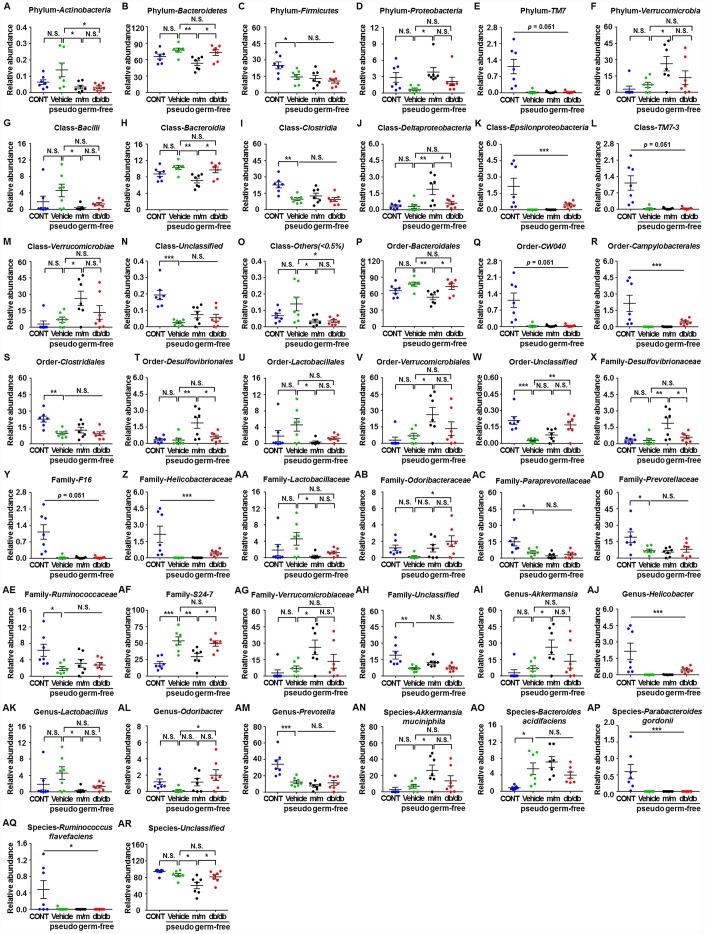
**Differences in the relative abundances of gut microbiota composition among pseudo-germ-free mice after transplantation from db/db and m/m mice.** (**A**) Phylum *Actinobacteria* (F_3,24_ = 4.607, *P* < 0.05); (**B**) Phylum *Bacteroidetes* (F_3,24_ = 5.458, *P* < 0.01); (**C**) Phylum *Firmicutes* (F_3,24_ = 5.936, *P*<0.01); (**D**) Phylum *Proteobacteria* (F_3,24_ = 3.204, *P*<0.05); (**E**) Phylum *TM7* (Fisher’s exact test; *P*=0.051); (**F**) Phylum *Verrucomicrobia* (F_3,24_ = 4.348, *P* < 0.05); (**G**) Class *Bacilli* (F_3,24_ = 2.968, *P* = 0.0521); (**H**) Class *Bacteroidia* (_F3,24_ = 5.465, *P*<0.01); (**I**) Class *Clostridia* (F_3,24_ = 8.279, *P* < 0.001); (**J**) Class *Deltaproteobacteria* (F_3,24_ = 7.044, *P* < 0.01); (**K**) Class *Epsilonproteobacteria* (Fisher’s exact test; *P*<0.001); (**L**) Class *TM7-3* (Fisher’s exact test; *P* = 0.051); (**M**) Class *Verrucomicrobiae* (F_3,24_ = 4.348, *P* < 0.05); (**N**) Class *Unclassified* (F_3,24_ = 12.87, *P* < 0.001); (**O**) Class *Others* (<0.5%) (F_3,24_ = 4.388, *P* < 0.05); (**P**) Order *Bacteroidales* (F_3,24_ = 5.465, *P* < 0.01); (**Q**) Order *CW040* (F_3,24_ = 12.36, *P* < 0.001); (**R**) Order *Campylobacterales* (Fisher’s exact test; *P* < 0.001); (**S**) Order *Clostridiales* (F_3,24_ = 8.316, *P* < 0.001); (**T**) Order *Desulfovibrionales* (F_3,24_ = 7.044, *P* < 0.01); (**U**) Order *Lactobacillales* (F_3,24_ = 2.97, *P*=0.0520); (**V**) Order *Verrucomicrobiales* (F_3,24_ = 4.348, *P* < 0.05); (**W**) Order *Unclassified* (F_3,24_ = 9.449, *P*<0.001); (**X**) Family *Desulfovibrionaceae* (F_3,24_ = 7.044, *P* < 0.01); (**Y**) Family *F16* (F_3,24_ = 12.36, *P* < 0.001); (**Z**) Family *Helicobacteraceae* (Fisher’s exact test; *P* < 0.001); (**AA**) Family *Lactobacillaceae* (F_3,24_ = 3.018, *P*<0.05); (**AB**) Family *Odoribacteraceae* (F_3,24_ = 3.087, *P*<0.05); (**AC**) Family *Paraprevotellaceae* (F_3,24_ = 7.505, *P* < 0.01); (**AD**) Family *Prevotellaceae* (F_3,24_ = 4.557, *P* < 0.05); (**AE**) Family *Ruminococcaceae* (F_3,24_ = 4.012, *P* < 0.05); (**AF**) Family *S24-7* (F_3,24_ = 11.79, *P* < 0.001); (**AG**) Family *Verrucomicrobiaceae* (F_3,24_ = 4.348, *P* < 0.05); (**AH**) Family *Unclassified* (F_3,24_ = 7.609, *P* < 0.001); (**AI**) Genus *Akkermansia* (F_3,24_ = 4.348, *P* < 0.05); (**AJ**) Genus *Helicobacter* (Fisher’s exact test; *P* < 0.001); (**AK**) Genus *Lactobacillus* (F_3,24_ = 3.018, *P*<0.05); (**AL**) Genus *Odoribacter* (F_3,24_ = 3.087, *P* < 0.05); (**AM**) Genus *Prevotella* (F_3,24_ = 12.57, *P* < 0.001); (**AN**) Species *Akkermansia muciniphila* (F_3,24_ = 4.348, *P* < 0.05); (**AO**) Species *Bacteroides acidifaciens* (F_3,24_ = 6.558, *P* < 0.01); (**AP**) Species *Parabacteroides gordonii* (Fisher’s exact test; *P* < 0.001); (**AQ**) Species *Ruminococcus flavefaciens* (Fisher’s exact test; *P* < 0.05); (**AR**) Species *Unclassified* (F_3,24_ = 7.505, *P* < 0.01). Data are shown as mean ± SEM values (n=7). *P<0.05, **P<0.01, ***P<0.001. CONT, control; NS, not significant; SEM, standard error of the mean.

A total of 44 gut bacteria at six levels (phylum, class, order, family, genus, and species) were significantly altered among the CONT, vehicle, m/m, and db/db groups (Figure 6A–6AR). Antibiotic-treated pseudo-germ-free mice showed a significant decrease in the levels of 19 gut bacteria compared to the control mice (Figure 6C, 6E, 6I, 6K, 6L, 6N, 6Q–6S, 6Y, 6Z, 6AC–6AE, 6AH, 6AJ, 6AM, 6AP, and 6AQ). However, db/db and m/m mice failed to elicit any changes in the levels of 17 gut bacteria in the pseudo-germ-free mice (Figure 6A, 6D, 6F, 6G, 6M, 6O, 6U–6W, 6AA, 6AB, 6AG, 6AI, 6AK, 6AL, 6AN, and 6AO). Intriguingly, gut microbiota transplants from the db/db or m/m mice further ameliorated or aggravated the alteration in the levels of the phylum *Bacteroidetes*, class *Bacteroidia*, class *Deltaproteobacteria*, order *Bacteroidales*, order *Desulfovibrionales*, family Desulfovibrionaceae, family S24-7, and species *Unclassified* (Figure 6B, 6H, 6J, 6P, 6T, 6X, 6AF, and 6AR).

### Abundances of the composition of fecal microbiota transplants in pseudo-germ-free mice at the phylum, class, order, family, genus, and species levels

**Figure 7:**
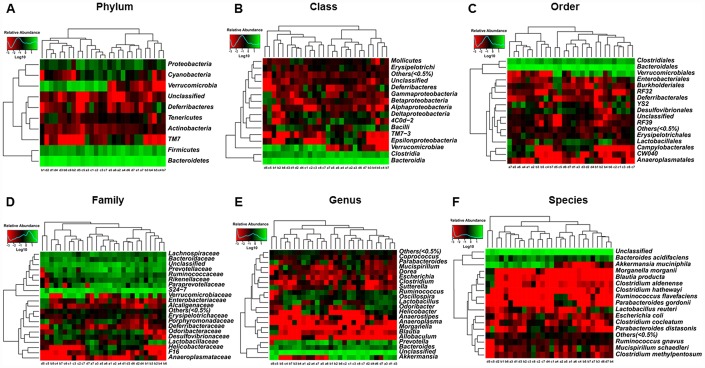
**Heat maps of the composition of the gut microbiota in pseudo-germ-free mice after transplantation from db/db and m/m mice.** (**A**) Phylum level; (**B**) Class level; (**C**) Order level; (**D**) Family level; (**E**) Genus level; (**F**) Species level.

Heat maps of the composition of fecal microbiota transplants in pseudo-germ-free mice at the phylum, class, order, family, genus, and species levels in the CONT, vehicle, m/m, and db/db groups are shown in Figure 7A–7F.

## DISCUSSION

Aging represents a predisposing factor for abnormalities in glucose metabolism and regulation [28]. It has been demonstrated that age-related glucose intolerance, and even hyperglycemia, might deleteriously influence the stability of glucose metabolism, finally causing the onset of T2DM [5]. Although various experimental models are used in the study of T2DM, db/db mice are confirmed as a rodent model that is genetically diabetic because of missense mutations in leptin receptors [24, 29], accompanied by high circulatory levels of leptin but lacking intracellular leptin action [30]. Given the fact that dysfunctional leptin signaling is highly associated with metabolic diseases, such as obesity and T2DM [31], we therefore adopted C57 BL/KS db/db male mice as a T2DM murine model. Although the mice used in this study were eight weeks old, we observed that the blood glucose, body weight, and water and food intake were significantly increased in db/db mice compared with the controls; these findings are likely similar to the clinical manifestations observed in individuals with aging phenotype.

It has been demonstrated in various studies that obese and overweight individuals typically exhibit the onset of systemic low-grade inflammation [9] and that the occurrence of chronic inflammation in individuals with obesity is suggested to promote the clinical progression of T2DM [32]. Furthermore, an imbalance in the composition of gut microbiota is a predisposing trigger for obesity-related inflammation. Abnormalities in the gut microbiota disrupt the gut mucosal barrier, thereby causing the release of toxins from the gut into the circulation, in spite of the ambiguous mechanisms of this release [33]. In addition, obesity-associated inflammation or even T2DM is more prevalent in patients with low α-diversity in gut microbiota compared to healthy individuals [34]. In the present study, we found that the Ace index, but not the Shannon index, was significantly lower in db/db mice compared to m/m mice, indicating decreased α-diversity with reduced species richness in the gut community of db/db mice. Moreover, the dissimilarity dots of β-diversity (PCA and PLS-DA) suggested that the microbiota composition was significantly altered between db/db and m/m mice. It is, therefore, likely that there is a tight relationship between the biologic profile of the gut microbiota and the development of obesity and T2DM, as well as other metabolic disorders.

16S rRNA gene sequencing has become an available approach for exploring the differential composition of gut microbiota [35–37]. In this study, db/db mice exhibited statistical alterations in 17 gut bacteria at six taxonomic levels (phylum, class, order, family, genus, and species), particularly characterized by a significant increase in *Verrucomicrobia* at six levels (phylum, class, order, etc.) and a significant decrease in *Bacteroidaceae* at the family, genus, and species levels, as well as *Prevotellaceae* at the family and genus levels. Several lines of evidence suggest that Gram-negative bacteria, including *Bacteroidetes and Proteobacteria*, could secrete LPS endotoxins, finally leading to metabolic endotoxemia and increased levels of *Bacteroidetes* and *Proteobacteria* in patients with T2DM [20]. Interestingly, *Bacteroidaceae* at the family, genus, and species levels and *Prevotellaceae* at the family and genus levels were decreased in db/db mice, which is inconsistent with the findings of previous studies. This discrepancy might be attributable both to the differential profiles in gut microbiota between humans and rodents and to the differential dietary intake, although detailed studies on the role of Bacteroidaceae and Prevotellaceae are required [38].

It has been identified that *Verrucomicrobia* as a beneficial bacterium widely distributed in the healthy human gut has the potential to control the inflammation process [39] and that reduced levels of *Verrucomicrobia* are indicative of an unstable microbial community or gut dysbiosis [40]. Furthermore, the relative abundance of *Verrucomicrobia* was shown to be decreased in individuals with T2DM [41]. Intriguingly, db/db mice possessed higher abundances of *Verrucomicrobia* at six phylogenetic levels in our study, implying that there is likely a positive feedback response to regulate the onset of inflammation. Altogether, an abnormal composition of the gut microbiota might directly, at least partially, contribute to low-grade inflammation, finally acting as a predisposing factor for the progression of obesity and T2DM.

Pseudo-germ-free mice induced by large doses of antibiotics were commonly used in fecal microbiota transplants in our previous studies and in other studies [35, 37, 42]. In the present study, vehicle-treated pseudo-germ-free mice showed significantly lower α-diversity after fecal bacteria transplantation with abnormalities in the Ace and Chao indices, suggesting a decreased number in bacteria. Additionally, β-diversity, including PCA and PLS-DA, revealed that the effects of fecal microbiota transplants from db/db and m/m mice to the host gut bacteria were obviously distinct. Furthermore, a total of 44 bacteria were altered at six levels after fecal microbiota transplantation. In vehicle-treated mice, 19 bacteria were significantly decreased, suggesting a deleterious effect induced by antibiotic treatment [43]. By contrast, the levels of phylum *Bacteroidetes*, class *Bacteroidia*, and order *Bacteroidales* were increased in the pseudo-germ-free mice after db/db fecal bacteria transplantation. These findings appear to be inconsistent with the results of gut microbiota in db/db mice. The physiological functions of gut microbiota are extremely complicated, and the interactions among the bacteria are fully determined. These complicated factors, combined with the use of antibiotics, make the changes in the gut microbiota more confusing. Although the specific mechanism of this difference is still unclear, these findings support the likelihood that alterations in the gut microbiota could ameliorate or aggravate the development of T2DM.

Overall, the findings described above could support the possibility that potential therapeutic strategies improving gut microbiota might exert beneficial effects for type 2 diabetes mellitus. Actually, increasing evidence has suggested that microbiota transplantation generated from animals or even humans into a germ-free environment may ameliorate or aggravate metabolic abnormalities. Intriguingly, a study on gut microbiota transplant from twins discordant for obesity into mice [44] has provided convincing evidence that the cross-talks between diet style and microbiota composition could influence the host biology. In addition, intestinal microbiota transplant from lean donors enhanced insulin sensitivity in individuals with metabolic syndromes [45], and that a clinical trial suggested that fecal microbiota transplantation has facilitating effects on recipient insulin sensitivity, energy expenditure and intestinal transit time [46].

There are several limitations in the study. First, a large number of mice are needed to diminish the discrepancy among the groups. Second, we did not adopt db/db mice at different age stages, especially the elderly. Third, clinical studies are required to figure out clinical and therapeutic implications, which would provide alternative strategies in the treatment of T2DM.

In conclusion, the findings of this study strongly suggest that an abnormal composition of gut microbiota contributes to the pathogenesis and progression of T2DM. In addition to lifestyle changes and antidiabetic medication prescriptions [47, 48], regulating specific gut microbiota, including Bacteroidetes, Prevotellaceae, and Verrucomicrobia, may be an effective and available therapeutic strategy for T2DM.

## MATERIALS AND METHODS

### Animals

Eight-week-old C57 BL/KS db/db male mice (Lepr-KO/KO, *n* = 6), C57BL/KS male m/m mice (Lepr-WT/WT, *n* = 6), and C57BL/6J male mice (20–25 g, *n* = 40) were purchased from Beijing Vital River Laboratory Animal Technology (Beijing, China). All mice were housed under standardized conditions on a 12 h light/dark cycle, with *ad libitum* access to food and water. The laboratory conditions were maintained with a consistent temperature of 22°C ± 2°C and relative humidity of 60% ± 5%. The mice were allowed to acclimate for a week before the experiments commenced. All experimental protocols and animal handling procedures were conducted according to the recommendations in the *Guide for the Care and Use of Laboratory Animals*, published by the National Institutes of Health (Publications No. 80-23, revised in 1996). This study was approved by the Experimental Animal Committee of Tongji Hospital, Tongji Medical College, Huazhong University of Science and Technology (Wuhan, China).

### Measurements of metabolic parameters

The body weight and water and food intake of each mouse were measured during the experiments. Meanwhile, the fasting blood glucose levels were assessed from a tail vein blood sample using a OneTouch^®^ Ultra blood glucose meter.

### Pseudo-germ-free murine model

Based on our previous studies [35, 37, 49, 50], a pseudo-germ-free murine model was induced by large doses of broad-spectrum antibiotics (ampicillin 1 g/L, neomycin sulfate 1 g/L, and metronidazole 1 g/L) (Sigma-Aldrich Co., Ltd., USA) that were dissolved in drinking water and administered *ad libitum* to C57BL/6J mice for 14 consecutive days. The drinking solution was renewed every two days.

### Transplantation of fecal microbiota

The mice were placed in a clean cage with sterilized filter paper. Feces were collected immediately after defecation in a sterilized centrifuge tube. The filter paper was replaced for each mouse. Fecal samples were stored in a freezer at −80°C until transplantation to the pseudo-germ-free mice [37, 51]. Fecal microbiota was prepared by diluting 1 g of fecal samples, obtained from db/db or m/m mice, in 10 mL of sterile saline. The fecal material was suspended, and 0.2 mL of the suspension was guided by gavage into each mouse recipient for 14 consecutive days [42].

### 16S rRNA analysis of fecal samples

After collecting all of the fecal samples, we immediately transferred these samples (−4°C) to Beijing Genomics Institute (Shenzhen, China) before performing a 16S rRNA analysis. These procedures were completed within one day; this protocol was similar to the one reported in previous studies by our group [35–38, 49]. DNA extraction was performed using a TIANamp Stool DNA Kit (Tiangen Biotech, Beijing, China). Genomic DNA was amplified in 50 μL triplicate reactions with bacterial 16S rRNA gene-specific primers (V3-V4 region): 338F (5′-ACTCCTACGGGAGGCAGC-3′) and 806R (5′-GGACTACHVGGGTWTCTAAT-3′). The reverse primer contained a sample barcode, and both primers were connected using an Illumina sequencing adapter. Polymerase chain reaction (PCR) products were purified, and the concentrations were adjusted for sequencing using an Illumina MiSeq PE300 system. The original sequencing reads from the sample were sorted according to the unique barcodes, and the barcodes, linkers, and PCR primer sequences were removed. The resultant sequences were screened for quality, and 70 or more base pairs were selected for bioinformatics analysis. All sequences were classified using the National Center for Biotechnology Information BLAST and SILVA databases. Distance calculation, operational taxonomic units cluster, rarefaction analysis, and estimator calculation (α-diversity and β-diversity) were performed using the MOTHUR program [52].

### Statistical analysis

Data are shown as the mean ± standard error of the mean (SEM). *P* < 0.05 was considered to be statistically significant. Comparisons among groups were analyzed using one-way or two-way analysis of variance, followed by post hoc Tukey’s test and Student’s *t*-test using GraphPad Prism 7.0 (GraphPad Software, San Diego, CA, USA). Other analyzes, including data normality and statistical significance of relationships among independent variables of nonnormal distributions, were performed using Kolmogorov–Smirnov test, Mann–Whitney *U* test, Fisher’s exact test, and SPSS software version 21.0 (IBM Corp., Armonk, NY, USA). PCA and PLS-DA were performed to visualize the similarities and the discriminations in db/db and m/m mice.

## References

[1] Chang AM, Halter JB. Aging and insulin secretion. Am J Physiol Endocrinol Metab. 2003; 284:E7–12. 10.1152/ajpendo.00366.2002 12485807

[2] Hennekens CH, Pfeffer MA, Newcomer JW, Jellinger PS, Garber A. Treatment of diabetes mellitus: the urgent need for multifactorial interventions. Am J Manag Care. 2014; 20:357–59. 25181565

[3] Ingelfinger JR, Jarcho JA. Increase in the incidence of diabetes and its implications. N Engl J Med. 2017; 376:1473–74. 10.1056/NEJMe1616575 28402766

[4] Ogurtsova K, da Rocha Fernandes JD, Huang Y, Linnenkamp U, Guariguata L, Cho NH, Cavan D, Shaw JE, Makaroff LE. IDF Diabetes Atlas: global estimates for the prevalence of diabetes for 2015 and 2040. Diabetes Res Clin Pract. 2017; 128:40–50. 10.1016/j.diabres.2017.03.024 28437734

[5] Dhaliwal R, Rosen CJ. Type 2 diabetes and aging: A not so sweet scenario for bone. Horm Metab Res. 2016; 48:771–78. 10.1055/s-0042-117719 27728926

[6] Umpierrez GE, Pasquel FJ. Management of inpatient hyperglycemia and diabetes in older adults. Diabetes Care. 2017; 40:509–17. 10.2337/dc16-0989 28325798 PMC5864102

[7] Zheng Y, Ley SH, Hu FB. Global aetiology and epidemiology of type 2 diabetes mellitus and its complications. Nat Rev Endocrinol. 2018; 14:88–98. 10.1038/nrendo.2017.151 29219149

[8] Munshi MN. Cognitive dysfunction in older adults with diabetes: what a clinician needs to know. Diabetes Care. 2017; 40:461–67. 10.2337/dc16-1229 28325796

[9] van Greevenbroek MM, Schalkwijk CG, Stehouwer CD. Obesity-associated low-grade inflammation in type 2 diabetes mellitus: causes and consequences. Neth J Med. 2013; 71:174–87. 23723111

[10] Skyler JS, Bakris GL, Bonifacio E, Darsow T, Eckel RH, Groop L, Groop PH, Handelsman Y, Insel RA, Mathieu C, McElvaine AT, Palmer JP, Pugliese A, et al. Differentiation of diabetes by pathophysiology, natural history, and prognosis. Diabetes. 2017; 66:241–55. 10.2337/db16-0806 27980006 PMC5384660

[11] Leslie RD, Palmer J, Schloot NC, Lernmark A. Diabetes at the crossroads: relevance of disease classification to pathophysiology and treatment. Diabetologia. 2016; 59:13–20. 10.1007/s00125-015-3789-z 26498592

[12] Riobó Serván P. Obesity and diabetes. Nutr Hosp. 2013 (Suppl 5); 28:138–43. 24010754 10.3305/nh.2013.28.sup5.6929

[13] Frydrych LM, Bian G, O’Lone DE, Ward PA, Delano MJ. Obesity and type 2 diabetes mellitus drive immune dysfunction, infection development, and sepsis mortality. J Leukoc Biol. 2018; 104:525–34. 10.1002/JLB.5VMR0118-021RR 30066958

[14] Saad MJ, Santos A, Prada PO. Linking gut microbiota and inflammation to obesity and insulin resistance. Physiology (Bethesda). 2016; 31:283–93. 10.1152/physiol.00041.2015 27252163

[15] de Clercq NC, Frissen MN, Groen AK, Nieuwdorp M. Gut microbiota and the gut-brain axis: new insights in the pathophysiology of metabolic syndrome. Psychosom Med. 2017; 79:874–79. 10.1097/PSY.0000000000000495 28557822

[16] Isolauri E. Microbiota and Obesity. Nestle Nutr Inst Workshop Ser. 2017; 88:95–105. 10.1159/000455217 28346926

[17] Clavel T, Desmarchelier C, Haller D, Gérard P, Rohn S, Lepage P, Daniel H. Intestinal microbiota in metabolic diseases: from bacterial community structure and functions to species of pathophysiological relevance. Gut Microbes. 2014; 5:544–51. 10.4161/gmic.29331 25003516

[18] Meijnikman AS, Gerdes VE, Nieuwdorp M, Herrema H. Evaluating causality of gut microbiota in obesity and diabetes in humans. Endocr Rev. 2018; 39:133–53. 10.1210/er.2017-00192 29309555

[19] Barlow GM, Yu A, Mathur R. Role of the gut microbiome in obesity and diabetes mellitus. Nutr Clin Pract. 2015; 30:787–97. 10.1177/0884533615609896 26452391

[20] Cani PD, Bibiloni R, Knauf C, Waget A, Neyrinck AM, Delzenne NM, Burcelin R. Changes in gut microbiota control metabolic endotoxemia-induced inflammation in high-fat diet-induced obesity and diabetes in mice. Diabetes. 2008; 57:1470–81. 10.2337/db07-1403 18305141

[21] Wu X, Ma C, Han L, Nawaz M, Gao F, Zhang X, Yu P, Zhao C, Li L, Zhou A, Wang J, Moore JE, Millar BC, Xu J. Molecular characterisation of the faecal microbiota in patients with type II diabetes. Curr Microbiol. 2010; 61:69–78. 10.1007/s00284-010-9582-9 20087741

[22] Sedighi M, Razavi S, Navab-Moghadam F, Khamseh ME, Alaei-Shahmiri F, Mehrtash A, Amirmozafari N. Comparison of gut microbiota in adult patients with type 2 diabetes and healthy individuals. Microb Pathog. 2017; 111:362–69. 10.1016/j.micpath.2017.08.038 28912092

[23] Gerritsen J, Smidt H, Rijkers GT, de Vos WM. Intestinal microbiota in human health and disease: the impact of probiotics. Genes Nutr. 2011; 6:209–40. 10.1007/s12263-011-0229-7 21617937 PMC3145058

[24] Kobayashi K, Forte TM, Taniguchi S, Ishida BY, Oka K, Chan L. The db/db mouse, a model for diabetic dyslipidemia: molecular characterization and effects of Western diet feeding. Metabolism. 2000; 49:22–31. 10.1016/S0026-0495(00)90588-2 10647060

[25] Li Z, Wang W, Liu D, Guo Y. Effects of Lactobacillus acidophilus on gut microbiota composition in broilers challenged with Clostridium perfringens. PLoS One. 2017; 12:e0188634. 10.1371/journal.pone.0188634 29190649 PMC5708699

[26] Bermon S, Petriz B, Kajėnienė A, Prestes J, Castell L, Franco OL. The microbiota: an exercise immunology perspective. Exerc Immunol Rev. 2015; 21:70–79. 25825908

[27] Yuan M, Li D, Zhang Z, Sun H, An M, Wang G. Endometriosis induces gut microbiota alterations in mice. Hum Reprod. 2018; 33:607–16. 10.1093/humrep/dex372 29462324

[28] Szoke E, Shrayyef MZ, Messing S, Woerle HJ, van Haeften TW, Meyer C, Mitrakou A, Pimenta W, Gerich JE. Effect of aging on glucose homeostasis: accelerated deterioration of beta-cell function in individuals with impaired glucose tolerance. Diabetes Care. 2008; 31:539–43. 10.2337/dc07-1443 18083793

[29] Wang B, Chandrasekera PC, Pippin JJ. Leptin- and leptin receptor-deficient rodent models: relevance for human type 2 diabetes. Curr Diabetes Rev. 2014; 10:131–45. 10.2174/1573399810666140508121012 24809394 PMC4082168

[30] Drel VR, Mashtalir N, Ilnytska O, Shin J, Li F, Lyzogubov VV, Obrosova IG. The leptin-deficient (ob/ob) mouse: a new animal model of peripheral neuropathy of type 2 diabetes and obesity. Diabetes. 2006; 55:3335–43. 10.2337/db06-0885 17130477

[31] Lee YH, Hsu HC, Kao PC, Shiao YJ, Yeh SH, Shie FS, Hsu SM, Yeh CW, Liu HK, Yang SB, Tsay HJ. Augmented insulin and leptin resistance of high fat diet-fed APPswe/PS1dE9 transgenic mice exacerbate obesity and glycemic dysregulation. Int J Mol Sci. 2018; 19:E2333. 10.3390/ijms19082333 30096853 PMC6121904

[32] Bleau C, Karelis AD, St-Pierre DH, Lamontagne L. Crosstalk between intestinal microbiota, adipose tissue and skeletal muscle as an early event in systemic low-grade inflammation and the development of obesity and diabetes. Diabetes Metab Res Rev. 2015; 31:545–61. 10.1002/dmrr.2617 25352002

[33] Cani PD, Osto M, Geurts L, Everard A. Involvement of gut microbiota in the development of low-grade inflammation and type 2 diabetes associated with obesity. Gut Microbes. 2012; 3:279–88. 10.4161/gmic.19625 22572877 PMC3463487

[34] Wen L, Duffy A. Factors influencing the gut microbiota, inflammation, and type 2 diabetes. J Nutr. 2017; 147:1468S–75S. 10.3945/jn.116.240754 28615382 PMC5483960

[35] Yu F, Han W, Zhan G, Li S, Xiang S, Zhu B, Jiang X, Yang L, Luo A, Hua F, Yang C. Abnormal gut microbiota composition contributes to cognitive dysfunction in streptozotocin-induced diabetic mice. Aging (Albany NY). 2019; 11:3262–79. 10.18632/aging.101978 31123221 PMC6555457

[36] Zhan G, Hua D, Huang N, Wang Y, Li S, Zhou Z, Yang N, Jiang R, Zhu B, Yang L, Yu F, Xu H, Yang C, Luo A. Anesthesia and surgery induce cognitive dysfunction in elderly male mice: the role of gut microbiota. Aging (Albany NY). 2019; 11:1778–90. 10.18632/aging.101871 30904902 PMC6461176

[37] Zhan G, Yang N, Li S, Huang N, Fang X, Zhang J, Zhu B, Yang L, Yang C, Luo A. Abnormal gut microbiota composition contributes to cognitive dysfunction in SAMP8 mice. Aging (Albany NY). 2018; 10:1257–67. 10.18632/aging.101464 29886457 PMC6046237

[38] Han M, Wang C, Liu P, Li D, Li Y, Ma X. Dietary fiber gap and host gut microbiota. Protein Pept Lett. 2017; 24:388–96. 10.2174/0929866524666170220113312 28219317

[39] Zhang L, Qin Q, Liu M, Zhang X, He F, Wang G. Akkermansia muciniphila can reduce the damage of gluco/lipotoxicity, oxidative stress and inflammation, and normalize intestine microbiota in streptozotocin-induced diabetic rats. Pathog Dis. 2018; 76. 10.1093/femspd/fty028 29668928

[40] de Vos WM. Microbe Profile: Akkermansia muciniphila: a conserved intestinal symbiont that acts as the gatekeeper of our mucosa. Microbiology. 2017; 163:646–48. 10.1099/mic.0.000444 28530168

[41] Le Chatelier E, Nielsen T, Qin J, Prifti E, Hildebrand F, Falony G, Almeida M, Arumugam M, Batto JM, Kennedy S, Leonard P, Li J, Burgdorf K, et al, and MetaHIT consortium. Richness of human gut microbiome correlates with metabolic markers. Nature. 2013; 500:541–46. 10.1038/nature12506 23985870

[42] Ge X, Zhao W, Ding C, Tian H, Xu L, Wang H, Ni L, Jiang J, Gong J, Zhu W, Zhu M, Li N. Potential role of fecal microbiota from patients with slow transit constipation in the regulation of gastrointestinal motility. Sci Rep. 2017; 7:441. 10.1038/s41598-017-00612-y 28348415 PMC5428802

[43] Lange K, Buerger M, Stallmach A, Bruns T. Effects of antibiotics on gut microbiota. Dig Dis. 2016; 34:260–68. 10.1159/000443360 27028893

[44] Ridaura VK, Faith JJ, Rey FE, Cheng J, Duncan AE, Kau AL, Griffin NW, Lombard V, Henrissat B, Bain JR, Muehlbauer MJ, Ilkayeva O, Semenkovich CF, et al. Gut microbiota from twins discordant for obesity modulate metabolism in mice. Science. 2013; 341:1241214. 10.1126/science.1241214 24009397 PMC3829625

[45] Vrieze A, Van Nood E, Holleman F, Salojärvi J, Kootte RS, Bartelsman JF, Dallinga-Thie GM, Ackermans MT, Serlie MJ, Oozeer R, Derrien M, Druesne A, Van Hylckama Vlieg JE, et al. Transfer of intestinal microbiota from lean donors increases insulin sensitivity in individuals with metabolic syndrome. Gastroenterology. 2012; 143:913–6.e7. 10.1053/j.gastro.2012.06.031 22728514

[46] de Groot P, Scheithauer T, Bakker GJ, Prodan A, Levin E, Khan MT, Herrema H, Ackermans M, Serlie MJM, de Brauw M, Levels JHM, Sales A, Gerdes VE, et al. Donor metabolic characteristics drive effects of faecal microbiota transplantation on recipient insulin sensitivity, energy expenditure and intestinal transit time. Gut. 2019. [Epub ahead of print]. 10.1136/gutjnl-2019-318320 31147381 PMC7034343

[47] Milligan S. Combination therapy for the improvement of long-term macrovascular and microvascular outcomes in type 2 diabetes: rationale and evidence for early initiation. J Diabetes Complications. 2016; 30:1177–85. 10.1016/j.jdiacomp.2016.03.010 27149916

[48] Chapman S. Foot care for people with diabetes: prevention of complications and treatment. Br J Community Nurs. 2017; 22:226–29. 10.12968/bjcn.2017.22.5.226 28467244

[49] Zhang J, Bi JJ, Guo GJ, Yang L, Zhu B, Zhan GF, Li S, Huang NN, Hashimoto K, Yang C, Luo AL. Abnormal composition of gut microbiota contributes to delirium-like behaviors after abdominal surgery in mice. CNS Neurosci Ther. 2019; 25:685–96. 10.1111/cns.13103 30680947 PMC6515708

[50] Yang C, Fang X, Zhan G, Huang N, Li S, Bi J, Jiang R, Yang L, Miao L, Zhu B, Luo A, Hashimoto K. Key role of gut microbiota in anhedonia-like phenotype in rodents with neuropathic pain. Transl Psychiatry. 2019; 9:57. 10.1038/s41398-019-0379-8 30705252 PMC6355832

[51] Yang C, Fujita Y, Ren Q, Ma M, Dong C, Hashimoto K. Bifidobacterium in the gut microbiota confer resilience to chronic social defeat stress in mice. Sci Rep. 2017; 7:45942. 10.1038/srep45942 28368029 PMC5377462

[52] Sun H, Wang N, Cang Z, Zhu C, Zhao L, Nie X, Cheng J, Xia F, Zhai H, Lu Y. Modulation of microbiota-gut-brain axis by berberine resulting in improved metabolic Status in high fat diet-fed rats. Obes Facts. 2016; 9:365–78. 10.1159/000449507 27898425 PMC5644798

